# Nanoliposomal irinotecan with fluorouracil for the treatment of advanced pancreatic cancer, a single institution experience

**DOI:** 10.1186/s12885-018-4605-1

**Published:** 2018-06-27

**Authors:** Danielle C. Glassman, Randze L. Palmaira, Christina M. Covington, Avni M. Desai, Geoffrey Y. Ku, Jia Li, James J. Harding, Anna M. Varghese, Eileen M. O’Reilly, Kenneth H. Yu

**Affiliations:** 1 David M. Rubenstein Center for Pancreatic Cancer Research, Memorial Sloan Kettering Cancer Center, Weil Cornell Medical College, New York, NY USA; 2 Gastrointestinal Oncology Service, Memorial Sloan Kettering Cancer Center, 300 East 66th Street, New York, NY 10065 USA

**Keywords:** Pancreatic cancer, Nanoliposomal irinotecan, MM-398, Nal-IRI, 5-fluorouracil

## Abstract

**Background:**

Effective treatment options for advanced pancreatic cancer are finite. NAPOLI-1, a phase III randomized trial, demonstrated the efficacy of nanoliposomal irinotecan with fluorouracil/leucovorin (nal-IRI + 5-FU/LV) for the treatment of advanced pancreatic cancer following progression on gemcitabine-based chemotherapy. There are limited additional data on the safety and efficacy of nal-IRI + 5-FU/LV following FDA approval in October 2015. We examined the post-approval safety and effectiveness of nal-IRI + 5-FU/LV in advanced pancreatic cancer patients receiving treatment at Memorial Sloan Kettering Cancer Center.

**Methods:**

A retrospective chart review was conducted of all patients beginning treatment with nal-IRI + 5-FU/LV from October 2015 through June 2017. Using the electronic medical record and institutional database, information was extracted pertaining to demographics, performance status (ECOG), prior therapies, dose, duration of treatment, adverse events, progression free survival (PFS), overall survival (OS) and treatment response.

**Results:**

Fifty six patients were identified. Median progression free survival (PFS) was 2.9 months and median overall survival (OS) was 5.3 months. Patients with prior disease progression on irinotecan experienced PFS and OS of 2.2 and 3.9 mo, respectively. Patients without prior irinotecan exposure experienced significantly longer PFS (4.8 mo, *p* = 0.02) and OS (7.7 mo, *p* = 0.002), as did patients who received prior irinotecan without disease progression (PFS, 5.7 mo, *p* = 0.04; OS, 9.0 mo, *p* = .04). Progression on prior irinotecan was associated with greater lines of prior advanced disease chemotherapy (2 vs 1). Dose reductions (DR) were most frequently due to fatigue (42%) and diarrhea (37%), but were not associated with worse outcomes. In fact, patients with ≥1 DR experienced longer PFS (5.4 v 2.6 mo, *p = 0.035*). Sequential therapy with nab-paclitaxel + gemcitabine (nab-P + Gem) followed by nal-IRI + 5-FU/LV (*n* = 25) resulted in OS of 23.0 mo. Mutations in TP53 were associated with shorter PFS.

**Conclusions:**

These data support the safety and efficacy of nal-IRI + 5-FU/LV, reinforcing results of NAPOLI-1. Patients without disease progression on prior irinotecan fared significantly better than patients with progression, when treated with nal-IRI + 5-FU/LV. Sequential therapy with nab-P + Gem followed by nal-IRI + 5-FU/LV demonstrates encouraging median OS. These findings provide guidance for patients most likely to benefit from nal-IRI + 5-FU/LV.

**Electronic supplementary material:**

The online version of this article (10.1186/s12885-018-4605-1) contains supplementary material, which is available to authorized users.

## Background

Pancreatic ductal adenocarcinoma (PDAC) remains an intractable illness due to late stage of presentation, a propensity to metastasize, relative resistance to cytotoxic treatment and the lack of effective targeted agents. In 2017, an estimated 53,670 new cases of pancreatic cancer were diagnosed [1]. The majority of patients have either regional (11.5%) or distant (52%) spread at presentation. With a low 5-year survival rate of only 8.2%, PDAC ranks as the 3rd leading cause of cancer deaths, with an estimated 43,090 patient deaths in 2017. It is estimated that PDAC will rise to the second leading cause of cancer mortality by 2030 [2].

The treatment landscape for advanced PDAC has significantly changed since 2010. Randomized phase III trials have demonstrated significant survival benefits of FOLFIRINOX (folinic acid, 5-fluorouracil, irinotecan and oxaliplatin) [3] or nab-paclitaxel + gemcitabine (nab-P + Gem) [4] compared with the prior standard of care, single agent gemcitabine, for frontline treatment. Nanoliposomal irinotecan (nal-IRI) is a novel formulation of irinotecan, encapsulating drug molecules within long-circulating liposome-based nanoparticles with resulting favorable pharmacokinetic and biodistribution properties [5]. Recently, the randomized phase III NAPOLI-1 trial demonstrated significant survival benefit of nal-IRI with fluorouracil/leucovorin (nal-IRI + 5-FU/LV) compared with 5-FU alone after disease-progression on gemcitabine-based chemotherapy, progression-free survival (PFS) of 3.1 vs 1.5 months, respectively (*p = 0.0001*) and overall survival (OS) of 6.1 vs 4.2 months (*p = 0.012*). Nal-IRI received FDA approval on October 22nd, 2015.

Due to the aggressiveness of this disease, and, until recently, the dearth of effective therapies, the majority of patients receive only a single line of chemotherapy [6, 7]. With the current availability of several lines of active combination therapy, studies describing outcomes of sequential therapy are greatly needed. In particular, evidence for how to best integrate nal-IRI + 5-FU/LV into the treatment algorithm is needed and to understand the dosing schedule of the regimen. This retrospective, single institution analysis was conducted to address these questions.

## Methods

### Patients

A retrospective review was conducted of all consecutive patients with advanced PDAC who began receiving treatment with nal-IRI + 5-FU/LV at Memorial Sloan Kettering Cancer Center (MSKCC) and its regional care network between October 2015 and June 2017. The electronic medical record (EMR) was interrogated for patient demographics, performance status (ECOG), date of diagnosis, date of advanced disease diagnosis and carbohydrate antigen 19–9 (CA 19–9) level at baseline, tumor and germline genomic results, prior treatment details and duration, nal-IRI + 5-FU/LV starting dose, nal-IRI + 5-FU/LV dose reductions, nal-IRI + 5-FU/LV treatment duration, adverse events and survival. Tumor and germline genomics were performed under an IRB approved protocol (NCT01775072). The MSK-IMPACT somatic analysis utilizes targeted next generation sequencing (NGS) of pancreatic tumor tissue to interrogate a panel of 410–481 genes. Germline analysis on DNA obtained from normal peripheral blood utilizes NGS to interrogate a panel of 76 genes associated with hereditary cancer predisposition. This retrospective analysis was granted a research waiver by the MSKCC Institutional Review Board.

### Outcome measures

All treatment related adverse events (AEs) that occurred while patients were treated with nal-IRI + 5-FU/LV were collected. All AEs and SAEs were graded per National Cancer Institute Common Terminology Criteria for Adverse Events (NCI-CTCAE V4.0).

Patients were assessed every 8–12 weeks by computed tomography (CT). Disease response was assessed using RECIST version 1.1 criteria. Response by change in CA 19–9 level was recorded. Date of disease progression on nal-IRI + 5-FU/LV treatment and date of death were recorded.

### Statistical analysis

Descriptive statistics were calculated as mean, median or percentages as appropriate. PFS was calculated from the time of first nal-IRI + 5-FU/LV administration to disease progression or death, whichever occurred first. Nal-IRI + 5-FU/LV OS was calculated from time of first nal-IRI + 5-FU/LV administration to death. Advanced disease OS was calculated from time of advanced disease diagnosis to death.

Patients without progression or death were censored at the last follow-up date as of November 2nd, 2017. Survival curves and median survival were estimated using the Kaplan–Meier method. Survival curves were compared using Log-rank (Mantel-Cox) test or Log-rank test for trend. Hazard ratios were calculated using Log-rank test with 95% confidence intervals.

## Results

### Patient and tumor characteristics

During the accrual period, *N* = 56 patients with advanced PDAC received treatment with nal-IRI + 5-FU/LV at MSKCC. All patients who received one or more administrations of nal-IRI + 5-FU/LV were included in the analysis. The patient characteristics are listed in Table 1. The median age was 68 years, range 42 to 88 years. The significant majority of patients had metastatic disease at the time of treatment onset, with only two patients with locally advanced disease. The majority (79%) of patients had an Eastern Cooperative Oncology Group (ECOG) performance status of 0 or 1, the remainder (20%) had an ECOG performance status of 2. Nineteen patients had prior surgery and nine patients received prior radiation therapy. Although the majority of patients received at least one (36%) or more (58%) lines of chemotherapy prior to receiving nal-IRI + 5-FU/LV, 4 (7%) patients were treated with nal-IRI + 5-FU/LV in the frontline, metastatic setting after failure of gemcitabine-based chemotherapy in the adjuvant setting.

**Table 1:** Characteristics of patients and tumors

	*N = 56 (%)*
Median Age (years, range)	68 (42–88)
Gender	
Male	29 (52)
Female	27 (48)
ECOG Performance Status	
0	3 (5)
1	41 (73)
2	12 (21)
Primary tumor location	
Head	28 (50)
Body	11 (20)
Tail	12 (21)
Body and tail	5 (9)
Stage at start of treatment	
III	2 (4)
IV	54 (96)
Metastatic sites	
Liver	41 (73)
Peritoneum	16 (29)
Lung	15 (27)
Distant lymph nodes	18 (32)
Other	10 (18)
Number of metastatic sites	
1	29 (52)
2	8 (14)
3 or more	16 (29)
Prior lines of advanced disease therapy	
0	4 (7)
1	20 (36)
2	21 (38)
3 or more	11 (20)

### Dosing and drug delivery

The majority of patients (70%) started nal-IRI + 5-FU/LV treatment with a dose of nal-IRI below the recommended 70 mg/m^2^ dose level, see Table 2. The median starting dose was 55 mg/m^2^. The choice of a lower starting dose at our institution is based on physician preference. Line of therapy and ECOG performance status were not factors associated with lower starting dose. The only statistically significant factor identified was age; the median age of patients starting at full dose was 63 versus 70 (*p = 0.01*). The majority of patients never experienced a dose reduction of nal-IRI, with 15 (27%) experiencing a single dose reduction and only 3 (5%) experiencing two dose reductions. Examining the sequence of chemotherapy regimens prior to nal-IRI + 5-FU/LV treatment, the vast majority followed one of two patterns. The most common sequence was treatment with either 5-FU based chemotherapy, typically FOLFIRINOX or FOLFOX, followed by gemcitabine-based chemotherapy, typically single agent gemcitabine or nab-P + Gem, or the inverse (referred to going forward as **Sequence 1**). Twenty-six (46%) experienced this pattern of treatment, followed by nal-IRI + 5-FU/LV treatment in the 3rd line or later. The second most common sequence, received by 25 (45%) patients, was treatment with gemcitabine-based chemotherapy, typically either gemcitabine alone or nab-P + Gem in the frontline or adjuvant setting followed by nal-IRI + 5-FU/LV treatment in the 2nd line (referred to going forward as **Sequence 2**). A small number of patients, 3 (5%), received nab-P + Gem followed by Gem/capecitabine (Cap), followed by nal-IRI + 5-FU/LV in the 3rd line. Two (4%) patients received sequential treatment that did not fit any of these patterns due to participation in clinical trials.

**Table 2:** Dosing, dose reductions and sequencing of nal-IRI + 5-FU/LV

	*N = 56 (%)*
Starting nal-IRI dose (mg/m2)	
< 50	23 (41)
55	9 (16)
60	7 (13)
70	17 (30)
Dose reductions (#)	
0	38 (68)
1	15 (27)
2	3 (5)
Treatment sequencing	
FOLF (IRIN) OX ← → (nab-P) + Gem → nal-IRI + 5-FU/LV	26 (46)
nab-P + Gem → nal-IRI + 5-FU/LV	25 (45)
nab-P + Gem → Gem/Cap → nal-IRI + 5-FU/LV	3 (5)
other	2 (4)

### Efficacy

**Fig. 1:**
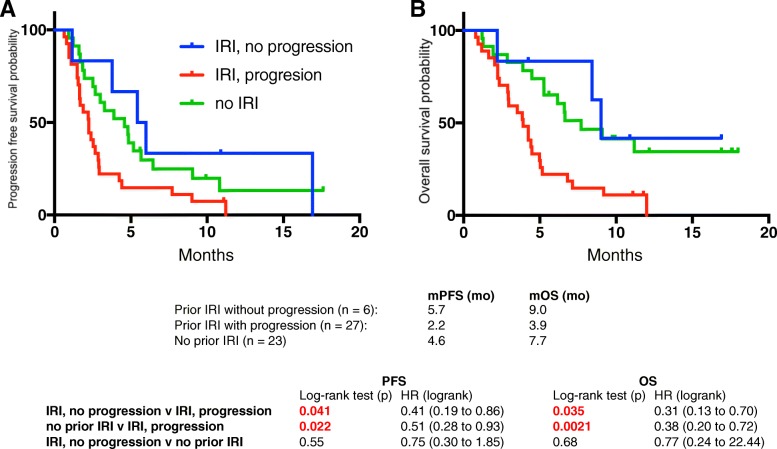
PFS (**a**) and OS (**b**) of patients receiving nal-IRI + 5-FU/LV based on prior irinotecan (IRI) based chemotherapy. Patients were classified based on whether their disease had not progressed on prior IRI-based chemotherapy (IRI, no progression), had progressed on prior IRI-based chemotherapy (IRI, progression), or had not received any prior IRI-based chemotherapy (no-IRI)

**Fig. 2:**
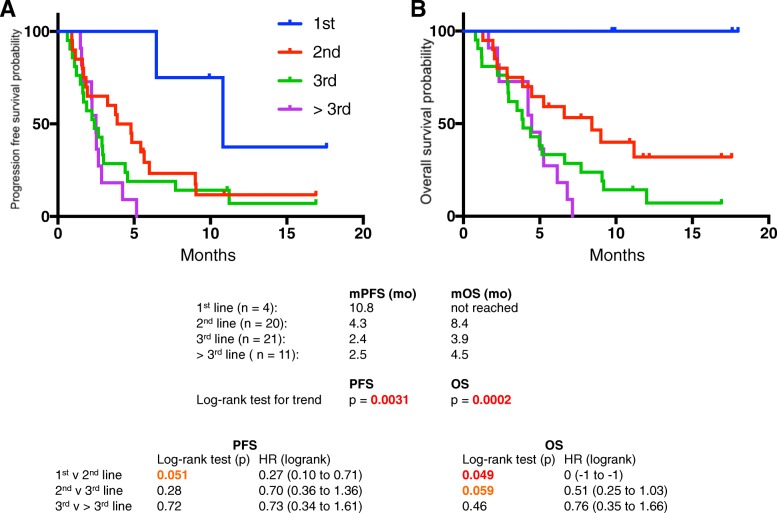
PFS (**a**) and OS (**b**) of patients receiving nal-IRI + 5-FU/LV based on line of therapy. Patients were classified based on the line of advanced-disease chemotherapy when nal-IRI + 5-FU/LV was administered

For the entire cohort of *N* = 56 the median PFS was 2.9 months and the median OS was 5.3 months (Table 3). Three patients had a PR (5%) and 23 (41%) had SD per RECIST. Ten patients (18%) experienced > 50% reduction of CA 19–9 at maximal response compared to baseline. Patients were classified based on whether they received irinotecan (*N* = 33, 59%) in prior lines of chemotherapy, or not (*N* = 23, 41%). Of patients receiving prior irinotecan, patients were further divided into those whose disease progressed on prior irinotecan-based chemotherapy (*N* = 27, 48%), or not (*N* = 6, 11%). The latter generally were patients who presented initially with locally advanced disease who completed a course of FOLFIRINOX chemotherapy in the neoadjuvant setting without disease progression before moving on to surgery or radiation therapy. By contrast, patients whose disease progressed on prior irinotecan-based chemotherapy typically received FOLFIRINOX as front-line therapy for metastatic disease. Patients receiving nal-IRI + 5-FU/LV after progressing on prior irinotecan-based chemotherapy experienced significantly shorter PFS and OS compared with patients not previously treated with irinotecan (PFS, 2.2 v 4.6 mo, *p = 0.022*; OS, 3.9 v 7.7 mo, *p = 0.0021*), and also when compared with patients previously treated with irinotecan without progression (PFS, 2.2 v 5.7 mo, *p = 0.041*; OS, 3.9 v 9.0 mo, *p = 0.035*) (Fig. 1). Importantly, patients with progression on prior irinotecan-based chemotherapy typically received nal-IRI + 5-FU/LV in a later line of therapy (median, 3rd-line) compared with the other sub-groups. Looking specifically at line of advanced disease therapy, there was a significant trend to longer PFS (*p = 0.0031*) and OS (*p = 0.0002*) for patients receiving nal-IRI + 5-FU/LV in earlier lines of therapy, compared with later (Fig. 2).

**Table 3:** Overall efficacy and response to treatment with nal-IRI + 5-FU/LV

	*N = 56 (%)*
PFS (median, mo)	2.9
OS (median, mo)	5.3
Response rate	
Partial response	3 (5)
Stable disease	23 (41)
Progressive disease	23 (41)
Not evaluabe	7 (13)
CA 19–9 response (maximal response/baseline)	
> 1	19 (34)
0.5 to 1	15 (27)
< 0.5	10 (18)
not evaluable	10 (18)
not measurable	2 (4)
Advanced disease, OS (median, mo)	
All	24.2
Sequence 1	25.5
Sequence 2	23.0
nab-P + Gem → Gem/Cap → nal-IRI + 5-FU/LV	28.6
other	23.0

ECOG performance status at start of nal-IRI + 5-FU/LV treatment was not significantly associated with PFS or OS. Twenty percent of patients in our cohort began treatment with an ECOG performance status of 2. This contrasts with patients treated with nal-IRI + 5-FU/LV in the NAPOLI-1 trial, where only 8.5% of patients began treatment with equivalent Karnofsky performance status of 70 or worse [8]. Starting dose of nal-IRI was also not significantly associated with survival, however, dose-reduction of nal-IRI was. Increasing numbers of dose reductions were associated with increased PFS (*p = 0.016*). There was also a trend to increased OS, though this did not meet statistical significance (*p = 0.073*). Comparing patients with or without any dose reductions, PFS was 5.4 v 2.6 mo (*p = 0.035*), OS was 7.1 v 4.5 mo (not significant, *p = 0.1226*).

**Fig. 3:**
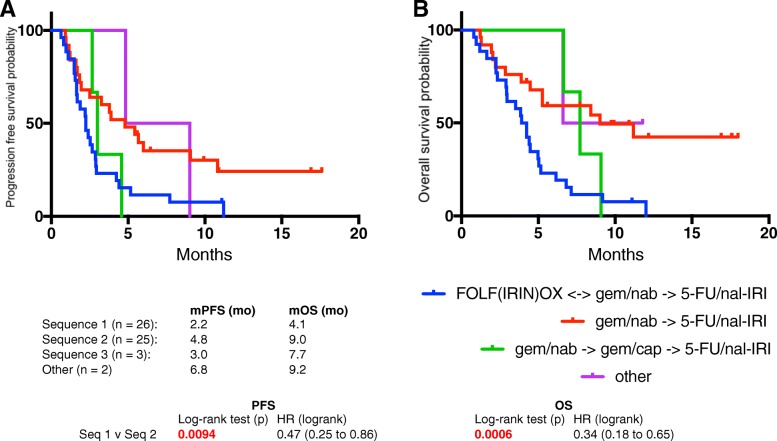
PFS (**a**) and OS (**b**) of patients receiving nal-IRI + 5-FU/LV based on treatment sequence. Patients were classified based on common treatment sequences utilized

Treatment sequences were significantly associated with survival (Fig. 3). Patients receiving Sequence 1 experienced significantly shorter PFS (2.2 v 4.8 mo, *p = 0.0094*) and OS (4.1 v 9.0 mo, *p = 0.0006*) compared with Sequence 2. OS from the time of advanced disease diagnosis was analyzed. Median OS from time of documentation of stage III or IV disease was 24.2 mo for all patients receiving nal-IRI + 5-FU/LV. OS was similar across all sequences of treatment (Table 3). A sequence of particular interest was Sequence 2, with patients receiving frontline Gem with or without nab-P, followed by nal-IRI + 5-FU/LV. Median OS was 23.0 mo.

### Safety

Patients were evaluated for toxicity through history and physical exam, complete blood count, and comprehensive metabolic panel. Treatment was discontinued at the discretion of treating physician due to toxicity or progression of disease. The number of dose reductions and attributed reasons for dose reductions are detailed in Table 4. Of the 20 total dose reductions, the most common reasons were for fatigue and diarrhea. Some dose reductions were attributed to multiple reasons. Adverse and serious adverse events are detailed in Table 5. Compared to the pivotal NAPOLI-1 trial, overall toxicity was comparable. There were lower rates of grade 3 or 4 toxicities seen in the MSKCC patient cohort across all of the most common toxicities observed, likely due in part to the lower median starting dose administered.

**Table 4:** Dose reductions and attributed reasons for dose reductions of nal-IRI + 5-FU/LV

Number of dose reductions	*N = 18 (%)*
1	15 (83)
2	3 (17)
Reason attributed for dose	
Fatigue	8 (44)
Diarrhea	8 (44)
Nausea	2 (11)
Neutropenia	2 (11)
Anorexia	2 (11)
Abdominal cramping	1 (6)
Ageusia	1 (6)
Not defined	1 (6)

**Table 5:** Adverse events and serious (grade 3 or 4) adverse events reported

	MSKCC	
Treatment	nal-IRI + 5-FU/LV	
Patients	56	
toxicities	any grade (%)	grade 3/4 (%)
Nausea	33 (59)	2 (4)
Vomiting	18 (32)	2 (4)
Diarrhea	35 (63)	1 (2)
Fatigue	45 (80)	1 (2)
Anorexia	32 (57)	0 (0)
Neutropenia	16 (29)	1 (2)
Anemia	50 (89)	10 (18)

### Tumor and germline genomics

Somatic with or without germline genomic results were available for 41 (73%) patients. The most commonly somatic gene mutations identified in the present study are similar to those identified in previously conducted, large genomic studies, and in similar proportions [9–13]. Activating mutations in KRAS were the most commonly identified, found in 83% of patients, followed by inactivating mutations in TP53 (66%), CDKN2A (29%) and SMAD4 (27%) (Additional file 1: Table S1). Germline mutations associated with cancer susceptibility were identified in 21% of patients, a frequency in-line with what our group has recently published in a large patient cohort. [14] BRCA2 was mutated in 3 patients. Although KRAS mutation status was not associated with PFS, TP53 mutation status was associated with significantly shorter PFS (2.2 v 6.0 mo, *p = 0.039*) (Additional file 1: Figure S1). There was a trend to shorter PFS in patients with mutations in SMAD4 and CDKN2A, however, neither of these differences reached statistical significance. Mutations in the four most common somatic genes were not associated with overall survival from the time of advanced disease diagnosis in this cohort. Germline mutations, including those in BRCA1 and BRCA2, were also not associated with differences in PFS or advanced disease overall survival, although the numbers of patients in these cohorts were exceedingly small.

## Discussion

Treatment options for advanced PDAC are expanding but nonetheless finite. Although PDAC remains a challenging disease, the last decade has seen the development of three new and effective combination chemotherapy regimens. The current study is the first report of post-approval, real-world analysis of nal-IRI + 5-FU/LV for the treatment of patients with advanced PDAC. This is also the first study reporting outcomes for patients in an era where two active, combination chemotherapy regimens, FOLFIRINOX and nab-P + Gem, are available for treating patients in the frontline/neoadjuvant settings, and an active, combination chemotherapy regimen, nal-IRI + 5-FU/LV, is available in the second-line setting.

The optimal sequencing of therapy remains undefined, and in practice, is largely defined by patient performance status, age, patient and physician preference. Molecular biomarkers, such as mutations in BRCA1/2 or microsatellite instability, to guide therapy are found in only a small minority of our patients [15]. For patients receiving FOLFIRINOX in the frontline setting, treatment with nab-P + Gem has been studied in a number of cohort studies. One of the largest was performed by the French AGEO (Association des Gastro-Entérologues Oncologues), [16] which studied a similarly sized cohort (*N* = 57) as our current study. Portal and colleagues found reasonable PFS (5.1 mo), OS (8.8 mo) and an encouraging median OS of 18 months from the beginning of advanced disease therapy. For patients receiving nab-P + Gem chemotherapy in the frontline setting, a number of 5-FU-based chemotherapy regimens have been studied. Chiorean and colleagues performed a retrospective analysis of patients enrolled in the pivotal MPACT study to evaluate 2nd therapy received [6]. In patients who received 2nd-line therapy, primarily 5-FU-based, after frontline nab-P + Gem, overall survival was 12.8 mo. The most common 5-FU-based regimens administered contained oxaliplatin. Irinotecan-based chemotherapy was uncommon, and none of these individuals received nal-IRI + 5-FU/LV.

Before the approval of nal-IRI + 5-FU/LV, the most common regimens for treatment after failure of gemcitabine-based chemotherapy were FOLFIRI and FOLFOX. The activity of FOLFIRI has been studied in a number of single arm studies. In one of the largest such studies, Zaniboni and colleagues found PFS and OS of 3.2 and 5 mo, respectively [17]. No randomized studies have been performed to support the efficacy of FOLFIRI in the 2nd line. Two randomized studies investigating the activity of 5-FU and oxaliplatin combinations report conflicting results. The randomized phase III CONKO-003 trial demonstrated a benefit of OFF, a 5-FU and oxaliplatin regimen commonly administered in Europe, compared to 5-FU alone, with PFS of 2.9 v 2.0 mo (*p = 0.019*), respectively, and OS of 5.9 v 3.3 mo (*p = 0.010*), respectively [18]. By contrast, the PANCREOX trial demonstrated no benefit of mFOLFOX6 compared with 5-FU, with PFS of 3.1 v 2.9 mo, and surprisingly a detriment in OS, 6.1 v 9.9 mo (*p = 0.02*) [19]. A single randomized phase II study has compared second line therapy with FOLFIRI to FOLFOX [20]. Both regimens performed similarly, with PFS of 1.9 and 1.4 mo, respectively, and OS of 3.8 and 3.4 mo, respectively. Overall survival from beginning of frontline therapy was 10.8 mo for both groups. A recent meta-analysis performed by Sonbol and colleagues comparing second-line therapies concluded that although both oxaliplatin and irinotecan improved PFS compared with 5-FU alone, only irinotecan appeared to improve OS [21].

Nal-IRI is a liposomal encapsulated formulation of irinotecan with favorable pharmacokinetic properties as demonstrated in preclinical [5] and preliminary clinical studies [22]. These results led to a phase II trial, [23] then the randomized phase III NAPOLI-1 trial. [8] NAPOLI-1 was a global study which enrolled 417 patients who previously received Gem-based chemotherapy. Patients were initially randomized to receive either nal-IRI monotherapy dosed at 120 mg/m^2^ every 3 weeks or 5-FU/LV monotherapy dosed at 2000 mg/m^2^/continuous infusion over 24 h weekly for 4 out of every 6-week cycle. A third arm, nal-IRI + 5-FU/LV, dosed at nal-IRI (70 mg/m^2^) with 5-FU/LV (2400 mg/m^2^/continuous infusion over 46 h), was added once the phase II dose of the combination was established. As previously discussed, NAPOLI-1 demonstrated both PFS (3.1 vs 1.5 mo, *p = 0.0001*) and OS benefit (6.1 vs 4.2 mo, *p = 0.012*) of nal-IRI + 5-FU/LV compared with 5-FU. In our current study, PFS (2.88 mo) for all patients treated with similar to that seen in the NAPOLI-1 study. A number of key factors were significantly associated with longer survival, including earlier line of therapy, non-progression on prior irinotecan-based chemotherapy, and dose-reductions while on treatment. In this real-world study, safety was comparable to that seen in the NAPOLI-1 study. The main toxicities seen were fatigue, gastrointestinal toxicities and cytopenias. The incidence of grade 3 or 4 toxicities was low. As part of the NAPOLI-1 study, patients found to be homozygous for the UGT1A1*28 allele were dosed at 50 mg/m^2^, then dose escalated to 70 mg/m^2^ in the absence of toxicity. Reassuringly, a separate safety analysis of the NAPOLI-1 study found that patients homozygous for the UGT1A1*28 allele (7/117) experienced similar treatment toxicity compared to those without [24]. Patients treated at our institution are not routinely tested for UGT1A1 genotype. Of note, the median starting dose administered of 55 mg/m^2^ is below that used in the NAPOLI-1 trial. This pre-emptive dose reduction represents real-world practice patterns and likely played a major role in the low rate of serious adverse events seen. Neither starting dose, nor dose reductions were associated with worse outcomes with regards to PFS or OS. This observation has been made in other regimens used for the treatment of advanced PDAC. For example, Ahn and colleagues found improved safety and promising efficacy when nab-P + Gem was administered at a lower, every other week, frequency [25]. Similarly, FOLFIRINOX with a variety of dose modifications is currently being studied. Two studies have found that dose reductions result in improved safety and similar [26] if not improved [27] efficacy. Lee and colleagues have developed a tool to optimize dose intensity for both toxicity and efficacy and applied their approach to FOLFIRINOX. [28] Studies to systematically examine this and other strategies to improve patient tolerance and outcomes should be undertaken.

With the increased prevalence of tumor somatic and patient germline sequencing, our ability to study the relationship between genomics and treatment response and survival will grow. A number of prior studies have studied gene mutations in KRAS, CDKN2A, TP53 and SMAD4 with regards to survival with mixed results. Hayashi and colleagues found that fewer numbers of mutations in these 4 key genes were associated with better prognosis [29]. Other studies have similarly found low p53 expression, [30] mutations in p16 and TP53, [31] and SMAD4 [32] as predictive of poor prognosis. While mutations in these genes did not correlate with overall survival from advanced stage disease in our patient cohort, there was a correlation between TP53 mutation status and PFS on nal-IRI + 5-FU/LV treatment, with a trend seen for CDKN2A and SMAD4. One preclinical study has previously demonstrated a relationship between TP53 mutation status and irinotecan sensitivity [33]. While no definitive conclusions can be drawn from a study of this size, our results suggest an interesting pharmacogenomic signal that merits further study and validation in larger, controlled patient cohorts.

The overall survival seen across all sequences of treatment was encouraging. In particular, patients receiving frontline nab-P + Gem followed by 2nd line nal-IRI + 5-FU/LV (*Sequence 2*) experienced an OS of 23.0 mo from the time of advanced disease diagnosis. Patients treated with FOLFIRINOX and nab-P + Gem prior to nal-IRI + 5-FU/LV (*Sequence 1*) also experienced excellent OS (25.5 mo), however, this was not significantly longer. This study represents the first published experience documenting survival in a patient population receiving treatment with access to all modern, FDA-approved chemotherapeutic agents. Given the toxicities experienced by some patients receiving FOLFIRINOX, the excellent survival seen in patients who did not receive FOLFIRINOX (*Sequence 2*) is encouraging and further studies to explore optimal sequencing are warranted. Overall advanced disease survival seen in our study compares favorably to OS reported with sequential nab-P + Gem then 5-FU-based chemotherapy (13.5 mo) [6] and sequential FOLFIRINOX and nab-P + Gem (18 mo). [16] Patient selection is likely a critical issue. Further studies are warranted to confirm the prolonged OS outcomes seen in this study. Patients who received nal-IRI + 5-FU/LV in the frontline metastatic setting experienced prolonged mPFS (10.82 mo) and mOS (not reached), however, the number of patients was very small. The use of nal-IRI for the frontline treatment of patients deserves further evaluation, and an ongoing study (ClinicalTrials.gov Identifier NCT02551991) will hopefully provide a definitive answer to this question.

As a single institution, retrospective analysis, the current study has limitations. Only patients without significant deterioration after prior gemcitabine-based chemotherapy and remained eligible for nal-IRI + 5-FU/LV chemotherapy were included. Patients treated at our tertiary referral center may not experience the same outcomes as patients treated in the community. Nevertheless, our results are encouraging and support continued utilization and study of the nal-IRI + 5-FU/LV regimen to treat patients with advanced pancreatic cancer, and to further optimize selection of patients most likely to benefit.

## Conclusions

This real-world study supports the findings of NAPOLI-1, demonstrating the safety and efficacy of nal-IRI + 5-FU/LV for the treatment of advanced PDAC following gemcitabine-based chemotherapy. Patients receiving nal-IRI + 5-FU/LV in earlier lines of therapy, and without irinotecan-refractory disease, experienced significantly longer PFS. Dose reductions were not associated with worse outcomes. Exploratory genetic predictors of response identified candidates which warrant validation. Promising OS was seen integrating nal-IRI + 5-FU/LV sequentially with active combination chemotherapy.

## Additional file

Additional file 1: **Figure S1.** PFS and tumor genomics. PFS was determined based on presence (mutant) or absence (wild type) of mutations in key tumor suppressor genes associated with PDAC: TP53 (A), SMAD4 (B) and CDKN2A (C). **Table S1.** Germline and somatic mutations identified using MSK-IMPACT sequencing. (PDF 165 kb)

## Abbreviations

AE: adverse event
CA 19–9: carbohydrate antigen 19–9
CT: computed tomography
DR: dose reductions
ECOG: Eastern Cooperative Oncology Group
EMR: electronic medical record
FOLFIRINOX: folinic acid, 5-fluorouracil, irinotecan and oxaliplatin
MSKCC: Memorial Sloan Kettering Cancer Center
nab-P + Gem: nab-paclitaxel + gemcitabine
nal-IRI + 5-FU/LV: nanoliposomal irinotecan with fluorouracil/leucovorin
NCI-CTCAE: National Cancer Institute Common Terminology Criteria for Adverse Events
NGS: next generation sequencing
OS: overall survival
PDAC: pancreatic ductal adenocarcinoma
PFS: performance status, progression free survival
SAE: serious adverse event
