# Supplementary material for: Nanoliposomal irinotecan with fluorouracil for the treatment of advanced pancreatic cancer, a single institution experience
Source: BMC Cancer. 2018 Jun 27;18:693. doi: 10.1186/s12885-018-4605-1 (PMC6020418; doi:10.1186/s12885-018-4605-1)
Supplement: Supplementary file 1 — Figure S1. PFS and tumor genomics. PFS was determined based on presence (mutant) or absence (wild type) of mutations in key tumor suppressor genes associated with PDAC: TP53 (A), SMAD4 (B) and CDKN2A (C). Table S1. Germline and somatic mutations identified using MSK-IMPACT sequencing. (PDF 165 kb) [file 12885_2018_4605_MOESM1_ESM.pdf]

**Supplemental Table 1**

| | ***N = 41 (%)*** |
|---|---|
| ***KRAS mutation*** | ***34 (83)*** |
| G12D | 15 (44) |
| G12R | 9 (26) |
| G12V | 5 (15) |
| G12C | 3 (9) |
| G12I | 1 (3) |
| Q61R | 1 (3) |
| ***TP53 mutation*** | ***27 (66)*** |
| ***CDKN2A mutation*** | ***12 (29)*** |
| ***SMAD4 mutation*** | ***11 (27)*** |
| ***Germline mutation*** | ***9 (21)*** |
| BRCA1 | 1 (11) |
| BRCA2 | 3 (33) |
| NF1 | 1 (11) |
| APC | 2 (22) |
| ATM | 1 (11) |
| MLH1 | 1 (11) |

**Supplemental Figure 1**

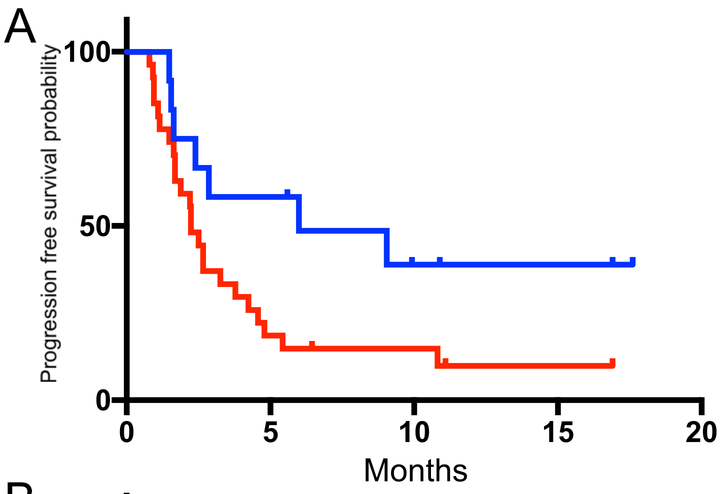


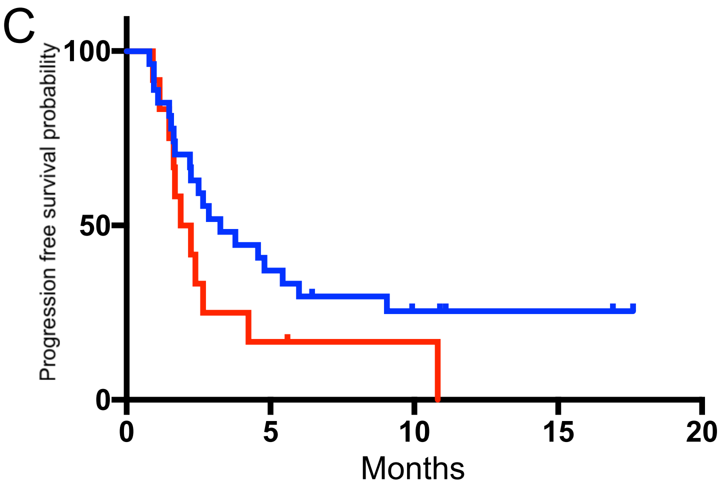


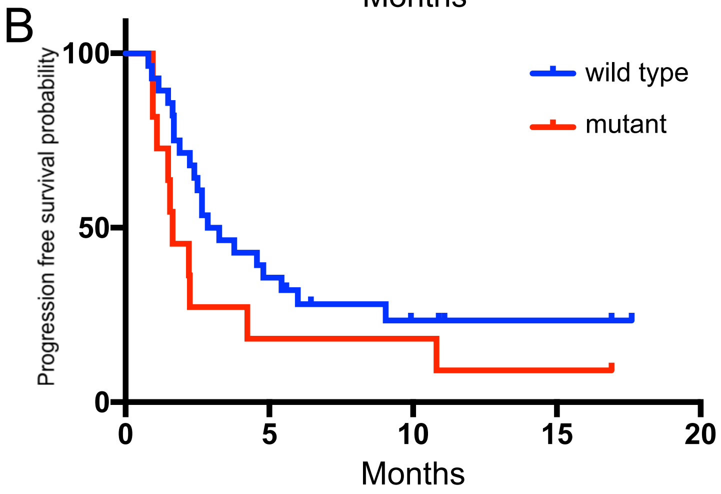


| Gene | status | mPFS (mo) | p | HR |
|---|---|---|---|---|
| TP53 | wild type | 6.0 | 0.039 | 0.43 (0.21 to 0.88) |
| | mutated | 2.2 | | |
| SMAD4 | wild type | 3.1 | 0.11 | 0.55 (0.23 to 1.31) |
| | mutated | 1.6 | | |
| CDKN2A | wild type | 3.3 | 0.11 | 0.56 (0.24 to 1.28) |
| | mutated | 2.1 | | |
